# Monascus orange and red pigments production by *Monascus purpureus* ATCC16436 through co-solid state fermentation of corn cob and glycerol: An eco-friendly environmental low cost approach

**DOI:** 10.1371/journal.pone.0207755

**Published:** 2018-12-10

**Authors:** Amira M. Embaby, Mohamed N. Hussein, Ahmed Hussein

**Affiliations:** 1 Institute of Graduate Studies and Research, Biotechnology department, Alexandria University, Alexandria, Egypt; 2 Department of Chemistry and Biochemistry, Texas Tech University, Lubbock, Texas, United States of America; University of Szeged, HUNGARY

## Abstract

The present study underlines a statistically optimized, low cost, effective approach for efficient co-valorization of two non-efficiently utilized, highly accumulated, raw agro-industrial wastes: corn cob and glycerol for co-production of natural biopigments: monascus orange and red pigments by the aid of *Monascus purpureus* strain ATCC 16436. A three step sequential, statistical modeling approach: one variable at a time (OVAT), Plackett-Burman design (PBD), and central composite design (CCD) was employed to optimize the production of monascus pigments using co-solid state fermentation of the two raw agro-industrial wastes. Corn cob among other carbon sources (e.g., rice grains, sugarcane bagasse, and potato peel) was the most appropriate substrate triggering co-production of orange and red monascus pigments; deduced from OVAT. Glycerol and inoculum size proved to impose significant consequences (P<0.05) on the production of monascus pigments as inferred from PBD. The optimal levels of inoculum size (12 x 10^11^ spores/mL) and glycerol (2.17 M) did achieve a maximal color value of 133.77 and 108.02 color value units/mL of orange and red pigments, respectively at 30 ^o^C after 10 days; concluded from CCD with an agitation speed of 150 rpm. Present data would underpin the large scale production of monascus pigments using the present approach for efficient exploitation of such biopigments in food, pharmaceutical and textile industries.

## Introduction

Uncontrolled usage of synthetic coloring agents (dyes) in food, pharmaceutical, and textile industries has posed critical environmental considerations and health issues [1]. Consequently, natural pigments (biopigments) are excellent alternates to replace such hazardous compounds. So far, plants and microorganisms are the major candidate hosts for a wide range of biopigments [2– 4]. However, microorganisms are superior over plants regarding certain issues, of prime importance are renewable sources for biopigments accompanied with massive production, controlled scaled up bioprocessing, easiness in genetic manipulation, fastly growing organisms, low, cost, effective growth and/or production media, simplicity in handling,no need for big land areas for growth like plants, etc [5]. Representative examples of microbial biopigments are zeaxanthin from *Staphylococcus aureus* and *Flavobacterium spp*., prodigiosin from *Serratia marcescens*, astaxanthin from *Sphingomonas spp*., pyocyanin blue from *Pseudomonas aeruginosa*, violacein from *Achrobacterium violaceum*, and monascus pigments from *Monascus spp*.[2–4, 6]. Monascus biopigments, red (rubropanctamine and monascorubramine), orange (monascorubrin and rubropunctatin), and yellow ankaflavin and monascin) ones, belong to azophilones [7, 8]. Reportedly, monascus biopigments are produced by nine species of the fungal genus *Monascus* such as *M*.*ruber* [9], *M*.*kaoliang*, *M*.*pilosus* [10], *M*. *sanguineus* [11], and *M*.*purpureus* [12–14].

As a rule of thumb, microbial strains belonging to the same species could exhibit various patterns concerning the yield of a traced bioproduct of a prime importance in the industrial or the pharmaceutical sectors [15]. Accordingly, this would necessitate the indispensable need for thorough selection of the most potent microbial strains to achieve the possible maximal yield of a traced bioproduct prior to the transfer from academia to industry.

Corn cob stands for 30% of the agricultural wastes derived from maize, the major plentiful cereal worldwide [16]. Inefficient utilization of such waste of high rich content of cellulose and hemicelluloses [17] would lead to its massive accumulation. Although, several researches did highlight its possible usage in biofuel production, but its surplus is in a remarkable continuous augmentation. Therefore, this in turn would pose potential environmental risks and health threats.

Glycerin (glycerol), a current industrial waste, is being produced as a large surplus from biodiesel production in the industrialized biodiesel countries worldwide. About 10% (wt/wt) of the biodiesel produced represents glycerol [18]. In spite of the involvement of glycerol in a wide range of industries, such as paint, soap, pharmaceuticals, food, and toothpaste, etc., novel usages are urgently needed to deal with the non-efficiently massive amounts of such waste [19].

The up to date review of literature contains a plethora of literature highlighting the production of monascus pigments from *Monascus spp*. through two main strategies: submerged state fermentation and solid state fermentation. The high cost encountered in submerged state fermentation [17] has greatly delimited the industrialization of such biopigments from *Monascus spp*. Conversely, solid state fermentation bears potential advantages over submerged state fermentation. Solid-state fermentation processes do have a substantial promise from the standpoint of environmental pollution that could be raised from downstream processing of submerged state fermentation [20].

The current review of literature does address the usage of a number of agro-industrial wastes with zero cost (e.g., rice bran, coconut oil cake, sugarcane bagasse, wheat bran, sesame oil cake, cassava powder, jackfruit seed powder, spent brewing grain, and orange processing waste) [21, 22] in the production medium for monascus biopigments from *Monascus spp*. Nevertheless, only one report highlighted the usage of corn cob in solid state fermentation for monascus biopigments production by the aid of one strain of *M*. *purpureus* namely *M*. *purpureus* KACC 42430 [17]. Reportedly, glycerol was used in both submerged state fermentation and solid state fermentation as a sole carbon source and an additive to solid wastes other than corn cob, respectively in the course of monascus pigments production [11, 23–26].

In the light of alleviating the capital cost included in natural pigment production and co-valorization of two non- efficiently utilized agro-industrial wastes (corn cob and glycerol), the objective of the present study is to optimize the co-production of orange and red monascus from *Monascus purpureus* strain ATCC16436 via co-solid state fermentation of corn cob and glycerol through statistical, sequential modeling approach. To the best of the authors’ knowledge, the present work is the first study addressing co-valorization of the two agro-industrial wastes: corn cob and glycerol as too cheap, efficient solid state fermentation approach for monascus orange and red pigments production with the aid of *Monascus purpureus* strain ATCC16436.

## Materials and methods

### Fungal strain

*Monascus purpureus* strain ATCC16436 was used in this study as a producer for monascus orange and red pigments.

### Carbon sources

The raw agro-industrial wastes corn cob, sugarcane bagasse, and potato peel were collected during the winter season of 2017. The collected wastes were milled into small fine particles (2 mm). Glycerol (99.9%) was purchased from local pharmacies in Alexandria City. However, rice grains were purchased from local markets in Alexandria City.

### Media

Potato dextrose agar [27] was used during the course of activation and short term preservation of the fungal strain. Four mL of modified minimal medium [28] (g/L:KNO_3_,50; MgSO_4_, 16.67and NaH_2_PO_4_, 25) were added to 30 g/L rice grains. The modified minimal medium was used as a core medium for monascus pigments biosynthesis in the initial steps of the optimization plan. The final pH of the medium was adjusted to be 3.5 unless otherwise stated.

### Seed culture

PDA plates were inoculated with the fungal spores. Then the inoculated plates were incubated in a static incubator (JSGI-100T, Korea) at 30 ^o^C for 7 days. A suspension of fungal spores was prepared using sterile water. Afterthat, the suspension was diluted and adjusted to be 2X10^4^ by hemocytometer.

### Extraction of monascus pigments

Monascus pigments were extracted according to a procedure reported previously with slight modifications [21]. At the end of the incubation period, 20 mL of 96% ethanol was added to the fermented cake. Then, the mixture was incubated for 2 h with an agitation speed of 180 rpm. After that, the mixture was filtered through Whatman paper #1.0. The filtrate was further centrifuged at 10, 000 rpm for 10 min. The supernatant was taken and kept at 4 ^o^C until being processed further.

### Determination of monascus pigment content

Monascus pigments production was evaluated in terms of orange and red pigments. Spectrophotometric measurements at 440 nm and 500 nm for orange and red pigments, respectively, were taken. Moreover, the content of the two pigments in the fungal cultures was determined in terms of color value unit /mL according to a method previously reported with slight modifications [29]. Color value was calculated as stated by Eqs (1 & 2) for orange and red pigments, respectively.

Colorvalueunit/mL=Abat440xdilutionfactorx1000/samplevolume(μL) (Eq 1)

Colorvalueunit/mL=Abat500xdilutionfactorx1000/samplevolume(μL) (Eq 2)

### Optimizing monascus pigments production

A three step sequential statistical strategy (OVAT, PBD, and CCD) was anticipated to optimize the production of monascus pigments by the aid of *M*. *purpureus* strain ATCC16436 under solid state fermentation conditions.

#### One variable at a time (OVAT) approach

Ordinarily, OVAT approach was employed to seek for the physicochemical key determinants that would impose significant effects on monascus pigments production. Herein, the tested key determinants were raw agro-industrial wastes instead of rice grains (i.e., sugarcane bagasse, corn cob, and potato peel), grams of the agro-industrial waste added in 250 mL Erlenmeyer flask, initial pH of the production medium, agitation speed, glycerol, and dextrose. Furthermore, all experimental runs of OVAT were performed in triplicates in 250 mL Erlenmeyer flasks under solid state fermentation conditions at 30 ^o^C.

#### Plackett–Burman Design (PBD)

Herein, PBD [30] was generated by Minitab software 17.3. Twenty experimental trials (fractional factorial design) were executed to monitor the linear effect of five independent variables, namely glycerol, inoculum size, KNO_3_, MgSO_4_, and incubation time on monascus pigments production (Table 1). Accordingly, the following polynomial equation of the first order Eq (3) was settled to portray the linear effect enforced by the five tested independent variables on the level of monascus pigments production.

Y=βo+∑βixi (Eq 3)

Where *Y* is the level of monascus pigments in terms of color value unit, *β*_*0*_ is the model intercept, *X*_*i*_ is the tested independent variable and *β*_*i*_ isthe co-efficient of the tested independent variable.

**Table 1:** Real-coded values of five independent variables in PBD along with experimental vs. predicted values of monascus pigments biosynthesized by *M*. *purpureus* strain ATCC16436.

Run #	Independent variable					Orange pigment Color value/mL		Red pigment Color value/mL	
	X1	X2	X3	X4	X5	Exp^a^.	Pred^b^.	Exp^a^.	Pred^b^.
1	1(1.5)	-1(2)	1(10)	1(3.33)	-1(7)	78.51	55.29	65.41	49.18
2	1(1.5)	1(6)	-1(2.5)	1(3.33)	1(18)	118.84	86.34	66.02	56.65
3	-1(0.375)	1(6)	1(10)	-1(0.83)	1(18)	21.28	21.39	10.51	14.05
4	-1(0.375)	-1(2)	1(10)	1(3.33)	-1(7)	20.89	14.39	17.50	16.48
5	1(1.5)	-1(2)	-1(2.5)	1(3.33)	1(18)	58.51	64.62	25.45	38.85
6	1(1.5)	1(6)	-1(2.5)	-1(0.83)	1(18)	75.51	72.87	60.04	47.71
7	1(1.5)	1(6)	1(10)	-1(0.83)	-1(7)	70.17	63.54	70.17	57.99
8	1(1.5)	1(6)	1(10)	1(3.33)	-1(7)	71.40	77.01	62.63	66.93
9	-1(0.375)	1(6)	1(10)	1(3.33)	1(18)	24.07	34.87	17.39	22.99
10	1(1.5)	-1(2)	1(10)	1(3.33)	1(18)	17.48	54.05	24.21	37.88
1	-1(0.375)	1(6)	-1(2.5)	1(3.33)	1(18)	22.58	45.43	10.17	24.00
12	1(1.5)	-1(2)	1(10)	-1(0.83)	1(18)	51.87	40.59	33.39	28.90
13	-1(0.375)	1(6)	-1(2.5)	1(3.33)	-1(7)	59.63	46.67	52.50	35.29
14	-1(0.375)	-1(2)	1(10)	-1(0.83)	1(18)	21.51	-0.32	10.84	-3.75
15	-1(0.375)	-1(2)	-1(2.5)	1(3.33)	-1(7)	31.72	24.96	24.38	17.49
16	-1(0.375)	-1(2)	-1(2.5)	-1(0.83)	1(18)	18.45	10.25	6.50	-2.73
17	1(1.5)	-1(2)	-1(2.5)	-1(0.83)	-1(7)	23.39	52.39	20.22	41.21
18	1(1.5)	1(6)	-1(2.5)	-1(0.83)	-1(7)	75.13	74.11	56.67	59.00
19	-1(0.375)	1(6)	1(10)	-1(0.83)	-1(7)	6.26	22.64	3.86	25.34
20	-1(0.375)	-1(2)	-1(2.5)	-1(0.83)	-1(7)	5.38	11.49	4.09	8.56

**X1**: Glycerol, **X2**: Inoculum size, **X3**: KNO_3_, **X4**: MgSO_4_,**X5**: Incubation time

Glycerin: in terms of M

Inoculum size was added in terms of number ofmilliliters of fungal spores suspension providing that each mL contains 2X10^4^ spores/mL

KNO_3_, MgSO_4_: in terms of (w/v)

a: experimental values

b: predicted values

#### Response surface methodology (RSM)

Lastly, each independent variable significantly influencing the yield of monascus pigments as deduced from PBD was subjected to a RSM approach in terms of central composite design (CCD) [31]. In this study, CCD was created by Minitab software 17.3. Herein, a CCD matrix of thirteen trials (Table 2) with two independent variables (glycerol and inoculum size) was employed to specify the optimal level of each key determinant along with the likely maximal level of response.

**Table 2:** Real-coded values of two independent variables in CCD along with experimental vs. predicted values of monascus pigments produced by *M*. *purpureus* strain ATCC16436.

Run#	Independent variable		Orange pigment Color value/mL		Red pigment Color value/mL	
	X1	X2	Exp^a^.	Pred^b^.	Exp^a^.	Pred^b^.
1	-1(1.25)	-1(6)	39.70	55.34	30.48	49.747
2	1(3.09)	-1(6)	79.99	74.11	89.22	72.36
3	-1(1.25)	1(10)	54.01	40.69	24.64	19.65
4	1(3.09)	1(10)	131.87	97.03	161.35	120.23
5	-1.414(0.872)	0(8)	31.85	26.24	30.42	15.81
6	1.414(3.47)	0(8)	54.52	79.34	66.44	102.92
7	0(2.17)	-1.414(5)	88.81	77.94	71.61	65.39
8	0 (2.17)	1.414(11)	53.71	83.79	49.86	77.95
9	0 (2.17)	0(8)	133.77	133.77	108.02	108.01
10	0 (2.17)	0(8)	133.77	133.77	108.02	108.01
11	0 (2.17)	0(8)	133.77	133.77	108.02	108.01
12	0 (2.17)	0(8)	133.77	133.77	108.02	108.01
13	0 (2.17)	0(8)	133.77	133.77	108.02	108.01

**X1**: glycerol in terms of M

**X2**: Inoculum size was added in terms of number of milliliters of fungal spores suspension providing that each mL contains 2X10^4^ spores/mL

a: experimental values

b: predicted values

CCD concludes all prospective interactions among studied independent variables that would enforce consequenceson theoutput through a second order polynomial Eq (4).

$$Y=\beta o+\sum_{i=1}^{2}\beta i\;xi+\sum_{i=1}^{2}\beta iixixi+\sum_{i=1i<J}^{2}\beta i\beta jxixj$$ (Eq 4)

Where *Y* is the level of monascus pigments in terms of color value (response), *x*_*1*_, *x*_*2*_, *x*_*3*,………_ *x*_*k*_ are the independent variables influencing the response, *β*_*0*_ is the model intercept, *βi* (i = 1, 2,…, k) is the linear estimate of the variable, *βii* (i = 1, 2,…, k) is the quadratic estimate of the variable, *βij* (i = 1, 2,…, k; *j* = 1, 2,…, k) is the cross interaction estimate of the variable and € is the random error. For statistical calculations, each independent variable *X* was coded as *Xi* according to Eq 5. where *X*_*i*_ is dimensional coded value of the independent variable, *xi* is the real value of this variable at this coded value, *x*_*o*_ is the real value of this variable at the center point (zero level) and *Δxi* is the step change value.

$$X_{i}=(xi-x_{o})/\Delta xi$$ (Eq 5)

All experimental trials in PBD and CCD were performed with an agitation speed of 150 rpm (New Brunswick incubator shaker, US) in 250 mL Erlenmeyer flasks under solid state fermentation conditions at 30 ^o^C.

### Statistical analysis

Minitab 17.3 software was used in this study to generate PBD-CCD matricesand carry out multiple linear and non-linear regression analyses. Moreover, Statistica 13.2 was used to depict the three dimensional surface plots. The standard error overlapping rule of Cumming et al was used to statistically analyze data of OVAT experiments [32].

## Results

**Fig 1:**
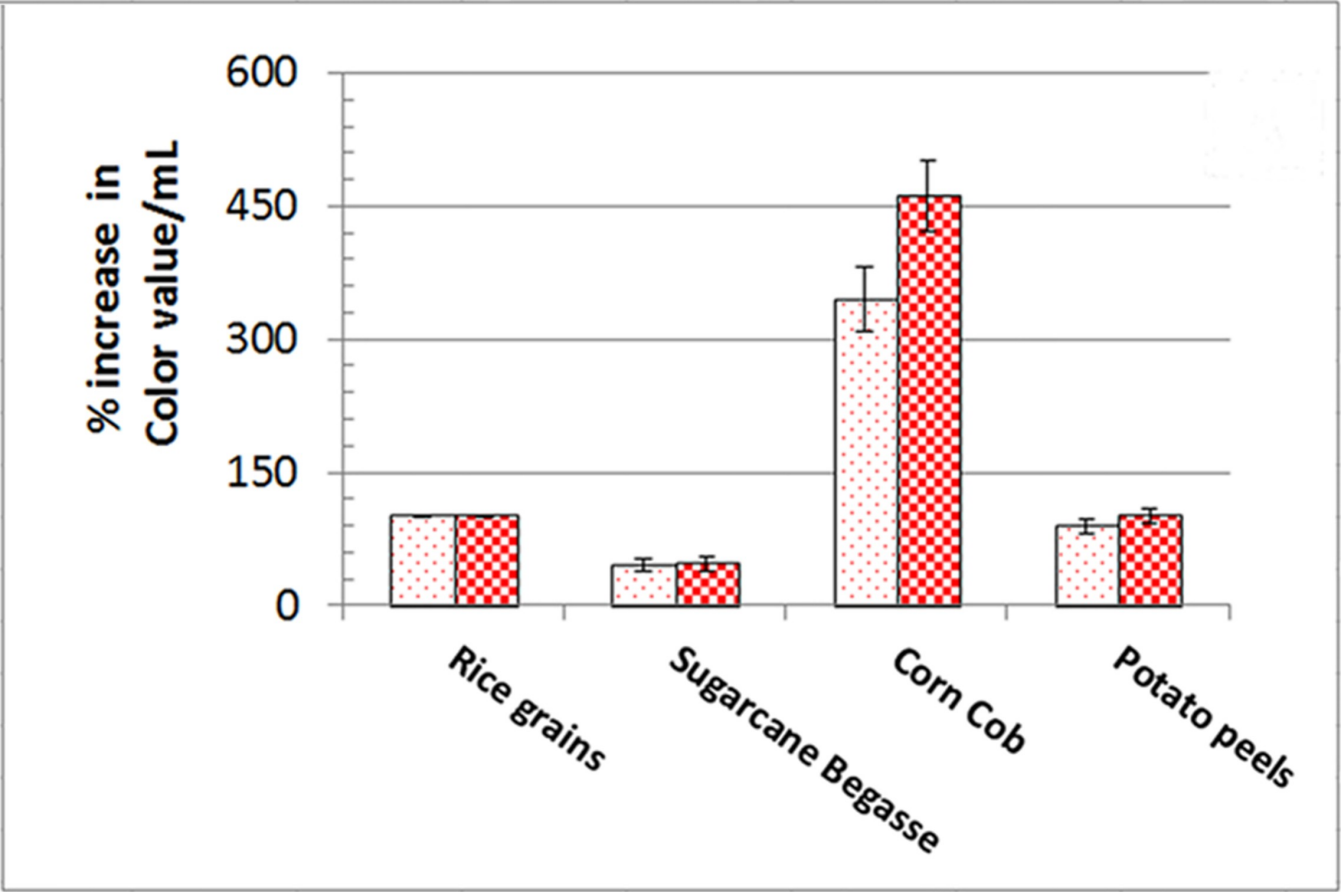
Effect of carbon source on orange and red monascus production. Dotted bars: orange moanscus pigments. Checkboard bars: red monascus pigments.

**Fig 2:**
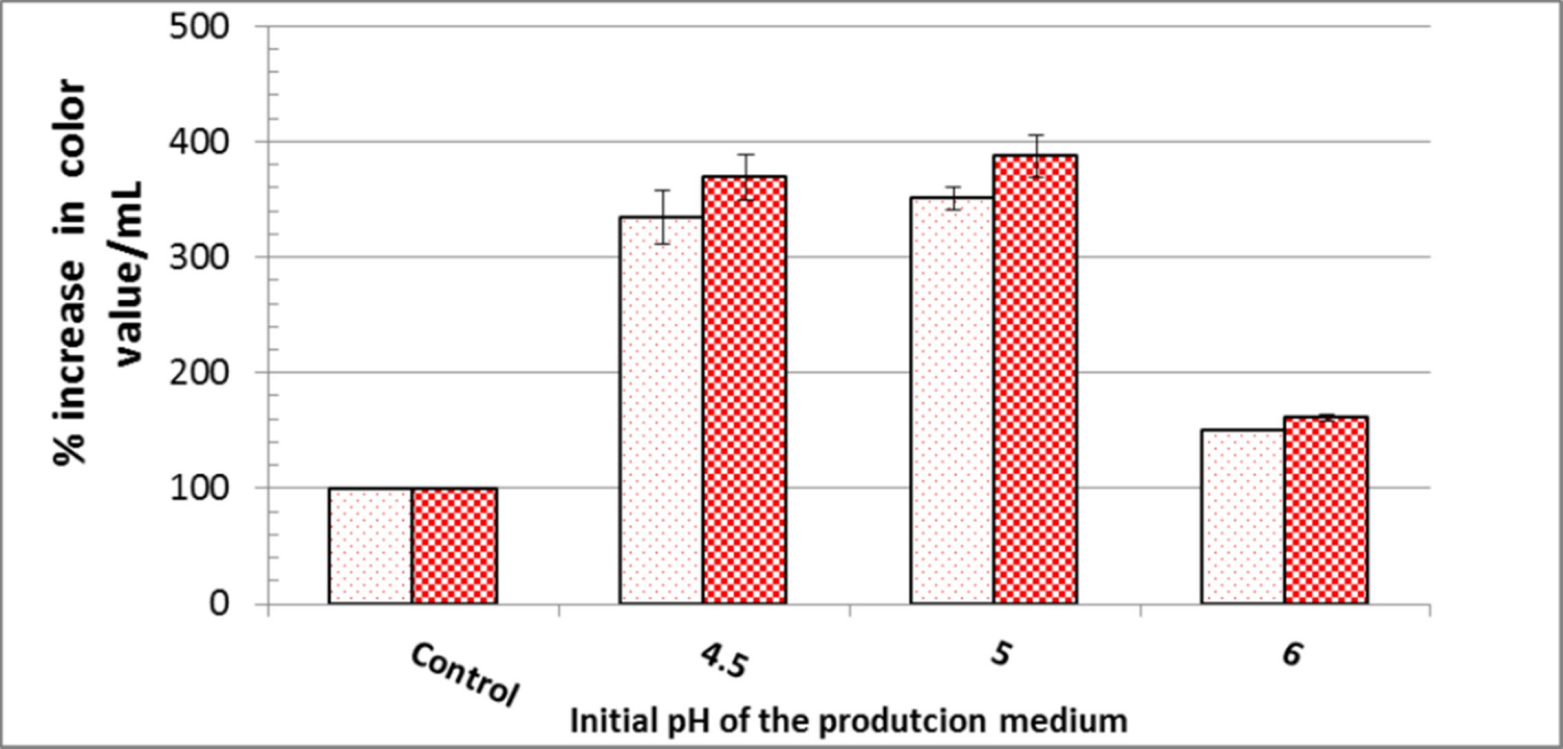
Effect of initial pH of the production medium on orange and red monascus pigments production. Dotted bars: orange moanscus pigments. Checkboard bars: red monascus pigments.

**Fig 3:**
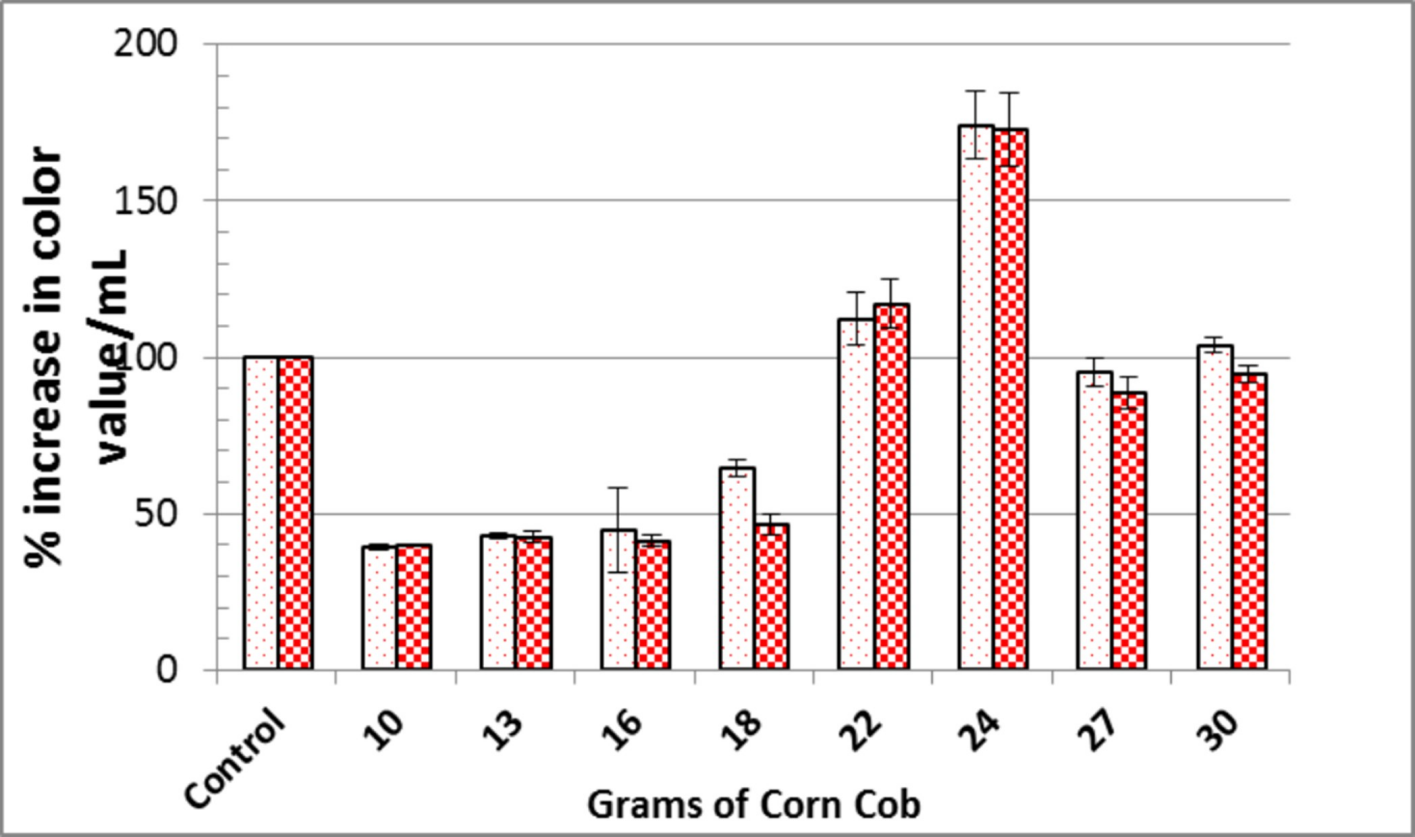
Effect of grams of corn cob added to the fermentation process on orange and red monascus pigments production. Dotted bars: orange moanscus pigments. Checkboard bars: red monascus pigments.

**Fig 4:**
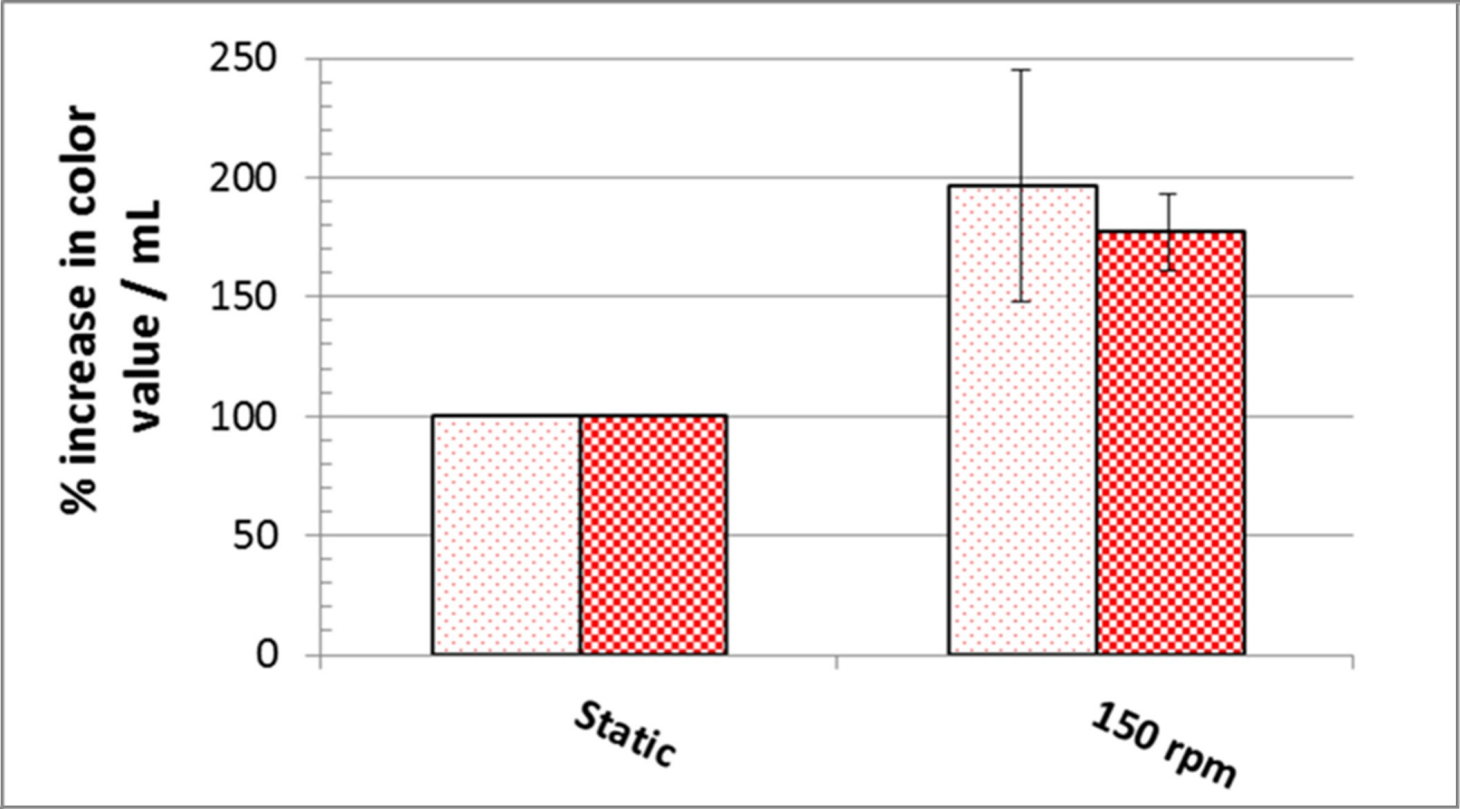
Effect of aeration on orange and red monascus pigments production. Dotted bars: orange moanscus pigments. Checkboard bars: red monascus pigments.

**Fig 5:**
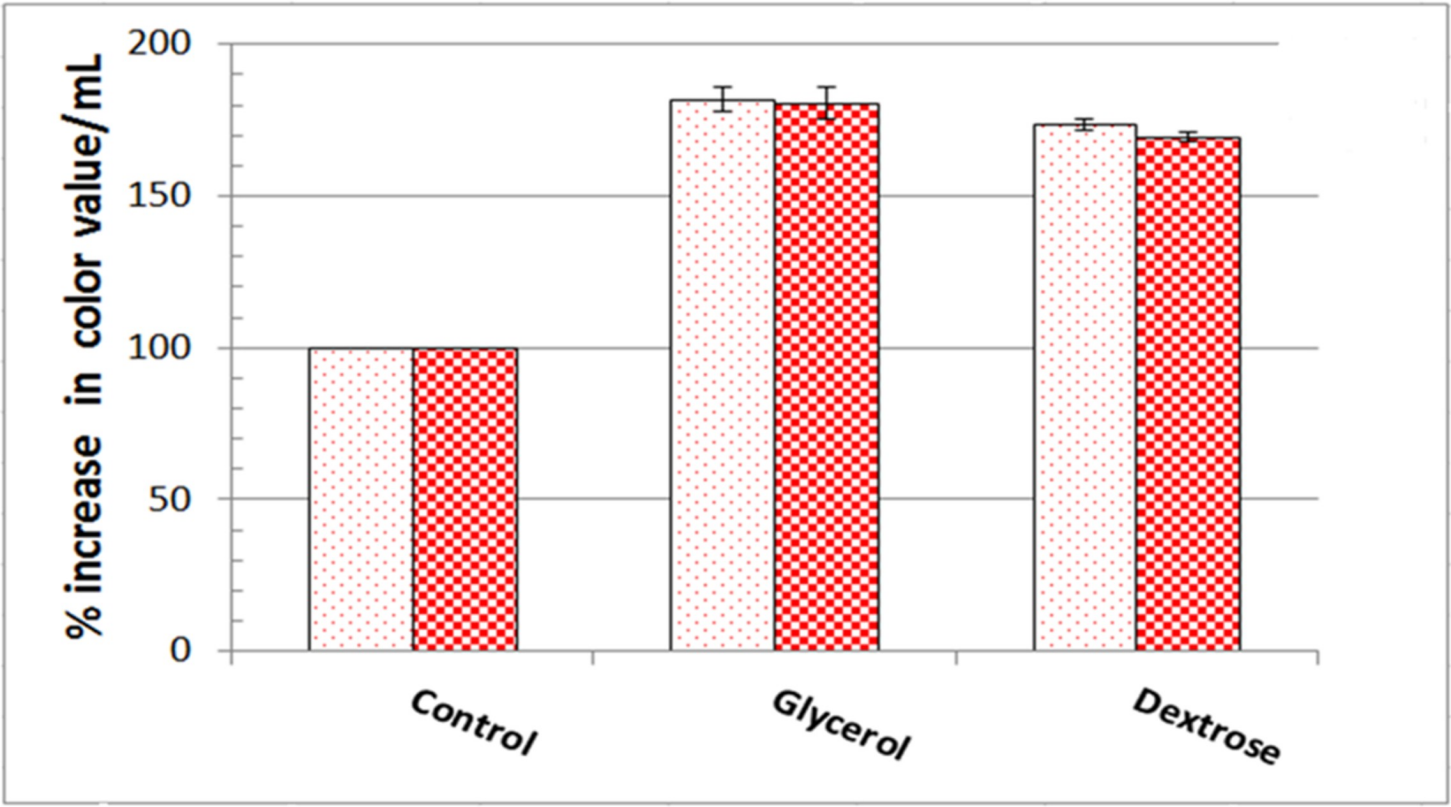
Effect of glucose and glycerol on orange and red monascus pigments production. Dotted bars: orange moanscus pigments. Checkboard bars: red monascus pigments.

Corn cob was the most appropriate raw agro-industrial waste triggering the highest significant levels (P <0.05) of orange and red monascus pigments by *M*. *purpureus* strain ATCC16436 when compared to the other three tested carbon sources: rice grains, sugarcane bagasse and potato peel (Fig 1). Corn cob did induce the co-production of orange and red pigments with an impressive significant percentage of increase 345% and 461.86%, respectively, when compared to those levels obtained in fungal cultures containing rice grains (Fig 1). With regard to the initial pH of the production medium, the highest significant levels of orange and red monascus pigments were achieved at pH 4.5–5.0 simultaneously with a marked significant decline in these levels at below and above this range (Fig 2). An imposed significant increase of 334.5% and 387.49% in the levels of orange and red pigments, respectively was noticed (Fig 2). The optimal amount of corn cob added to the production medium, triggering the highest significant levels of orange and monascus pigments, was 24 grams of this waste in 250 mL Erlenmeyer flask (Fig 3). This amount did succeed to elicit a significant increase of 174.14% and 172.96% in the levels of orange and red pigments concurrently, respectively (Fig 3). Pertaining to agitation speed, incubation of cultures with an agitation speed of 150 rpm did result in a significant enhancement in the levels of orange and red pigments: 196.38% and 177.06%, respectively, when compared to those levels of static cultures (Fig 4). The addition of dextrose and glycerol to the cultures separately did achieve significant elevations in the levels of orange and red pigments of 173.71% -181.93% and 169.54% - 180.67% concomitantly, respectively when compared to those levels of cultures lacking glycerol or dextrose (Fig 5). Based on data of OVAT experiments, corn cob, initial pH of the production medium of 4.5, glycerol and 150 rpm were selected as proper culture conditions that would promote the co-production of orange and red pigments.

Coded–real values of the PBD matrix along with experimental values versus predicted ones for the process outcome are displayed in Table 1 whilst, results of linear multiple regression are illustrated in Table 3. Experimental values of orange and red pigments in terms of color value unit did range from 5.38 to 118.84 and 3.86 to 70.17 that in turn did address the crucial role to perform PBD (Table 1).

**Table 3:** Multiple linear regression of PBD data for biosynthesis of monascus orange and red pigments by *Monascus purpureus* strain ATCC16436.

Model term	B-coefficient		t-value		P-value		% confidence	
	A	B	A	B	A	B	A	B
Intercept	43.629	32.09	9.613	10.039	1.52E-07*	8.9E-08*	99.99*	99.99*
X1	20.452	16.32	4.507	5.105	0.00049*	0.00016*	99.95*	99.98*
X2	10.859	8.89	2.393	2.783	0.03130*	0.014653*	96.87*	98.54
X3	-5.284	-0.51	-1.164	-0.159	0.26379	0.876157	-	-
X4	6.734	4.47	1.484	1.398	0.16001	0.184009	-	-
X5	-0.619	-5.65	-0.136	-1.766	0.893481	0.099243*	-	90.08*

A: *Significant P-value<0.05, R^2^:068, Adjusted R^2^: 056,P-value for the model = 0.0038

B: *Significant P-value≤ 0.1, R^2^:074, Adjusted R^2^: 064, P-value for the model = 0.001

**A**: ornage monascus pigments **B**: red monascus pigments

Multiple linear regression analysis of PBD did conclude that only glycerol and inoculum size significantly promote the co-production of orange and red pigments (Table 3). For orange pigments production, the model F- and P-values as elucidated from ANOVA were found to be 5.9 and 0.0038, respectively.Whilst, for red pigments production, the model F- and P-values as elucidated from ANOVA were found to be 7.78 and 0.0019, respectively. The significance of the model of orange pigments production was indicated by this F-value, while the model P-value reflects that the likelihood that this F-model value could take place due to noise is just 0.038%. However, the significance of the model of orange pigments production was indicated by this F-value, while the model P-value reflects that the possibility that this F-model value could take place due to noise is just 0.01%. Significance of co-efficient was evaluated by considering the P-value of the estimate. In addition, fitness of the model to explore the relevance between the response (outcome) and the significant independent variables most likely would be estimated by the small models P-values 0.0038 and 0.0019.

Glycerol and inoculum size did provoke positive significant influences on co-production of monascus orange and red pigments as deduced from results of linear multiple regression of the model.

**Fig 6:**
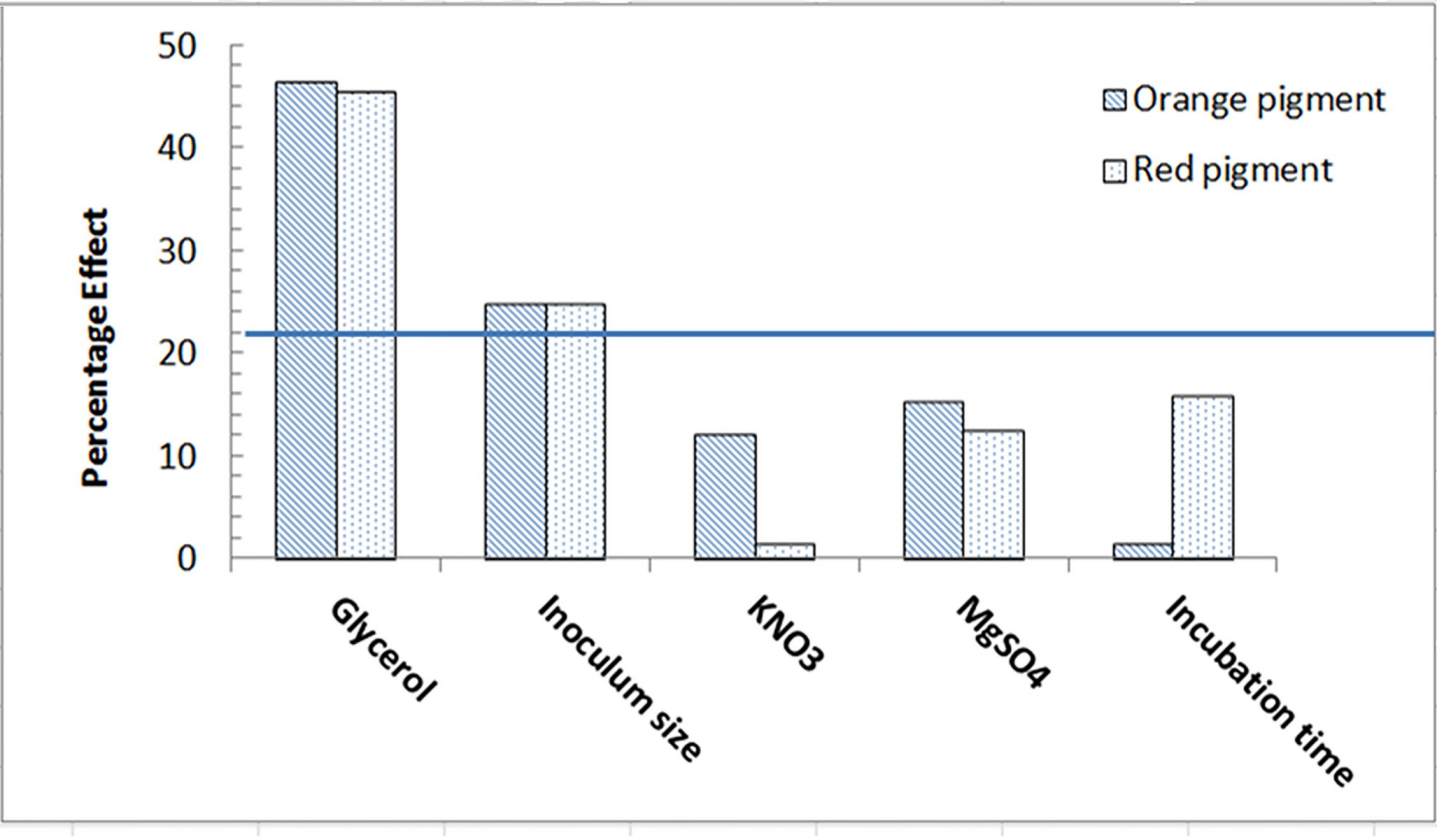
Pareto chart in a descending order concerning the significance of five tested independent variables on orange and red monascus pigments production. Solid line represents the level of significance(P<0.05).

Normally, demonstrating the significant consequences exerted by the tested independent variables on a process response could be easily achieved by depicting a Pareto chart. Here, a Pareto chart illustrated in Fig 6 was portrayed to present the order at which the tested five independent variables would influence the production of monascus pigments. Moreover, the full first order polynomial Eqs (6 and 7) in terms of coded values were established to explain the linear relevance between the independent variables and the response (orange and red pigments production), respectively as follows:

Y=43.629+20.45X1+10.859X2−5.28X3+6.73X4−0.6197X5 (Eq 6)

Y=32.09+16.32X1+8.39X2−0.51X3+4.47X4−5.65X5 (Eq 7)

The independent variables exhibiting non-significant influences on the process outcome were settled at intermediate level values (e.g., incubation time, KNO_3_ and MgSO_4_). Data derived from PBD revealed that glycerol (X1), inoculum size (X2) were specified as key determinants controlling the co-production of monascus orange and red pigments. Accordingly, these independent variables further underwent detailed analysis via RSM approach in terms of CCD. The real-coded values of the independent variables along with experimental versus predicted levels of orange and red pigments were displayed in Table 2, whereas, data of multiple non-linear regression were displayed in Table 4.

**Table 4:** Multiple non-linear regression of CCD data for biosynthesis of monascus orange and red pigments by *M*. *purpureus* strain ATCC16436.

Model term	B-coefficient		t-value		P-value		% confidence	
	A	B	A	B	A	B	A	B
Intercept	133.77	108.01	13.691	9.278	2.61E-06*	3.5E-05*	99.99*	99.99*
X1	18.78	30.79	2.431	3.346	0.045*	0.012*	95.5*	98.8*
X2	2.07	4.44	0.268	0.483	0.797	0.644	-	-
X1.X1	-40.51	-24.34	-4.889	-2.465	0.0018*	0.043*	99.82*	95.7*
X2.X2	-26.46	-18.18	-3.194	-1.841	0.015*	0.108	98.50*	-
X1.X2	9.39	19.49	0.859	1.4976	0.418	0.178	-	-

A: *Significant P-value<0.05, R^2^:0.84, Adjusted R^2^: 0.73, P-value for the model = 0.01

B: *Significant P-value<0.05, R^2^:0.76, Adjusted R^2^: 0.59, P-value for the model = 0.039

**A**: ornage monascus pigments **B**: red monascus pigments

ANOVA indicated that the model F-value and P-value were 7.45 and 0.01, respectively for orange pigment production. These values implied the significance of the model and the chance (1%) that this F-value might happen as a result of noise. On the contrary, the model F-value and P-value were 4.42 and 0.0381, respectively for red pigment production. These values indicated the significance of the model and the chance (3%) that this F-value might occur owing to noise. Models aptness is proved by the value of R^2^ (0.84) and R^2^ (0.76) that conferred the good concord between the experimental and the predicted values of the response (orange and red pigments, respectively). The second order polynomial Eqs (8 and 9) were fixed in terms of coded values to illustrate all probable interactions among the studied independent variables.

Y=133.77+18.78X1+2.07X2−40.511X1.X1−26.46X2.X2+9.39X1.X2 (Eq 8)

Y=108.01+30.79X1+4.44X2−24.34X1.X1−18.18X2.X2+19.49X1.X2 (Eq 9)

Multiple non-linear regressions showed that, the independent variables; glycerol and inoculum size did affect the co-production of the orange pigment in linear andquadratic interaction manners. Conversely, only the independent variable glycerol did influence the production of red pigment in linear and quadratic interaction manners.

After differentiation of both Eqs 8 & 9 to get the optimal predicted values of the independent variables X1 and X2 along with the maximal achieved predicted values of the two responses, the following two combination sets were obtained X1 = 0, X2 = 0 in terms of coded values to achieve a maximal level of orange and red pigments of 108 color value/mL and 133.76 color value/mL for red and orange pigments, respectively.

**Fig 7:**
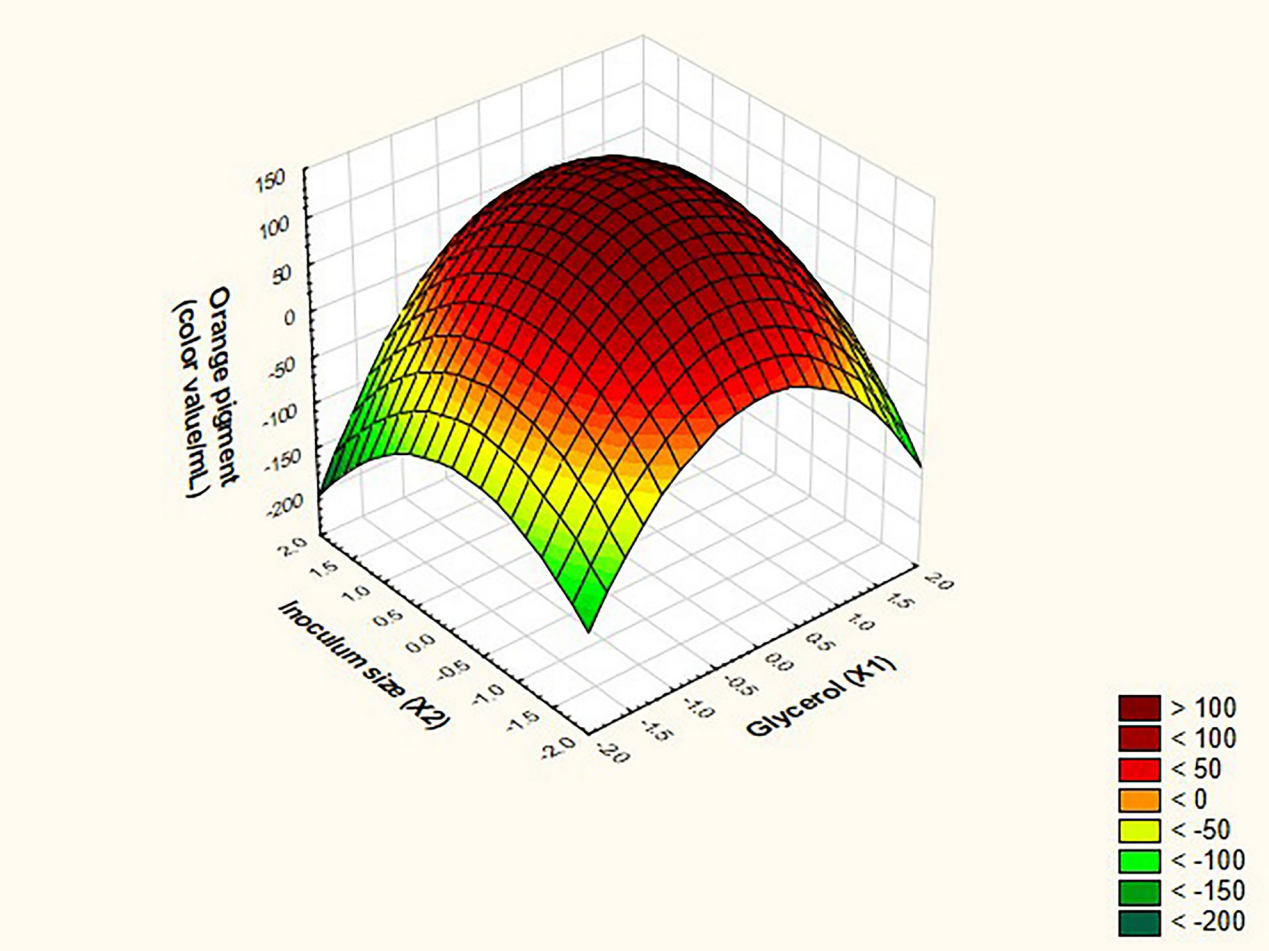
Three dimensional surface plots for the independent variables glycerol and inoculum size in relation to orange monascus pigments production.

**Fig 8:**
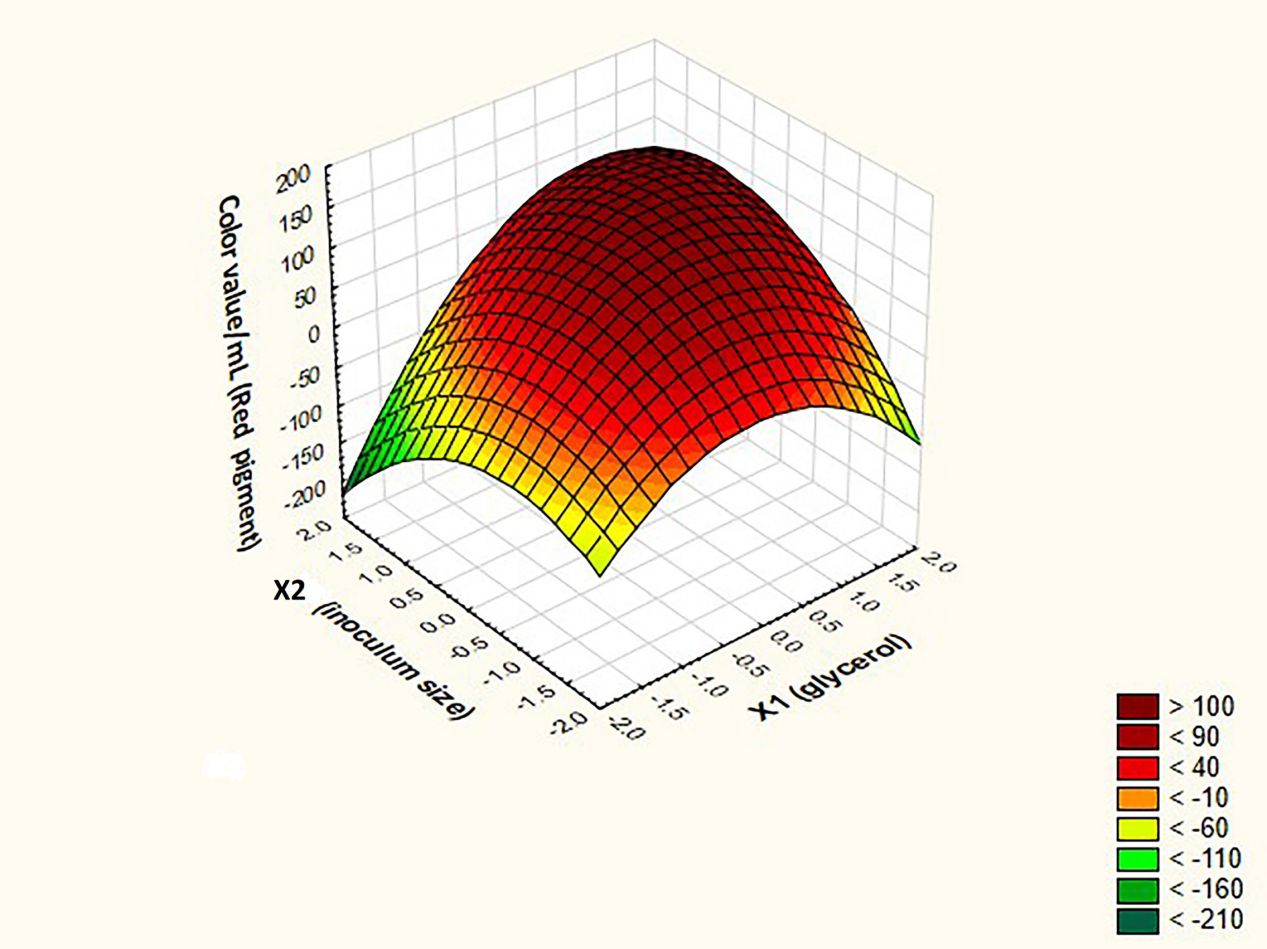
Three dimensional surface plots for the independent variables glycerol and inoculum size in relation to red monascus pigments production.

Further confirmation regarding the predicted optimal levels of X1 and X2 to achieve the maximal response from both red and orange pigments was obtained from the three dimensional surface plots, depicted in Figs 7 and 8. The maximal response of both pigments could be achieved at the center point of the design. Upon validating the predicted combination set of the independent variables at the center of the design in terms of real values (X1 = 2.17 M and X2 = 12X10^11^) in relation to the response in the laboratory, both models exhibited 100% validation.

## Discussion

Only one report did highlight the usage of corn cob as a solid low cost, effective substrate for monascus pigments production from merely one fungal strain of the genus *Monascus* namely *Monascus purpureus* KACC 42430. In the context of studying the influence of microbial strain biodiversity on monascus production, reducing the capital cost included in monascus pigments production, and maximizing monascus yield, the process of monascus red and orange pigments production was fully detailed optimized using statistical modeling approach via the fungal strain *Monascus purpureus* strain ATCC16436 through corn cob and glycerol co-solid state fermentation in this study.

Herein, the process of monascus production from *M*. *purpureus* strain ATCC16436 was subjected to a detailed three step statistical modeling approach; OVAT, PBD, and CCD. Statistical modeling is a robust approach towards optimization of the yield of outputs from various chemical and biological processes [33–35]. In this regard, the optimal level of each key determinant controlling the monascus pigments production from the candidate fungal strain was localized exactly to avoid the usage of extra amounts of medium components. This in turn would delimit the extra cost resulting from using extra amounts of medium components without real usage by the fungal strain.

With regard to the influence of fungal strain biodiversity on monascus pigments production, the comparison concerning yield of pigments from the two fungal strains: *M*. *purpureus* KACC 42430 and *M*. *purpureus* strain ATCC 16436 would not be justified yet, since, the units of pigments determinations from *M*. *purpureus* KACC 42430 upon growing on the corn cob substrate greatly differ from those derived from *M*. *purpureus* ATCC 16436 upon growing on the same substrate.

The highest levels of red and orange monascus pigments produced by *M*. *purpureus* strain ATCC16436 upon using corn cob as the main carbon source in the production medium of monascus pigments might be attributed to the nature of this waste with more remarkable rough textures when compared to the other two wastes; sugarcane bagasse and potato peel. The rough texture of corn cob might make this waste more preferential by *M*. *purpureus* strain ATCC16436 to potato peel and sugarcane bagasse to help facilitate the adhesion of fungal mycelia to corn cob waste in the course of solid state fermentation. Close adhesion of fungal mycelia to corn cob waste during solid state fermentation would facilitate bioavailability of cellulose and hemicellulose (main components of corn cob) [17, 36] to fungal cellulases and xylanases. As a consequence, the hydrolytic enzymes [37, 38] would degrade these complex carbohydrates with release of simple sugars that would in turn increase the fungal growth and the pigment production as well. The unique tight configuration of cellulose and hemicellulose in a lignin matrix in sugarcane bagasse might greatly hinder the fungal attack for cellulose and hemicellulose. Hence, the released simple sugars would be reduced with a reduction in the fungal growth rate and the pigments yield as well [36]. For potato peel, it has a high content of water [39] that makes this waste less hydrophobic when compared to corn cob. Consequently, the more hydrophobic nature of corn cob may encourage the close adhesion and penetration of fungal mycelia on the surface and the interior parts of the waste. Moreover, the low content of carbohydrates in the form of starch (7.8 g /100 g of potato peel) in potato peel [39] when compared to the relatively high content of cellulose (32.3–45.6%) and hemicellulose (39.8%) of corn cob [11] might be responsible for the low yield of monascus pigments from potato peel containing *M*. *purpureus* strain ATCC16436 cultures. The fully milled corn cob solid substrate does provide high surface area for fungal growth when compared to the other two wastes. Consequently, higher fungal growth rates would be associated with higher levels of pigments production.

In the meantime, the highest levels of monascus pigments achieved by *M*. *purpureus* strain ATCC16436 upon using corn cob when compared to rice grains would add an extra privilege to the usage of corn cob in solid state fermentation in this regard. This privilege is mainly confined to reducing the capital cost included in monascus pigment production, since rice grains are an edible crop not an agro-industrial waste.

The initial pH of the production medium is one the detrimental factor for the success of a bioprocess. Reportedly, there exists a discrepancy in the optimal initial pH of the monascus pigment production medium for numerous strains of *Monascus spp*. The discrepancy in the optimal pH of monascus pigments production could be attributed to the strain difference and the composition of the preferable production medium by the fungal strain. Unlike present finding of opimal pH of 4.5–5 for red and orange monascus pigments production from *M*. *purpureus* strain ATCC16436, the optimal pH of red monascus production from *M*. *purpureus* strain CCT3802 and *M*. *purpureus* strain MTCC 1090 was at the alkaline side (8–9) upon growing on synthetic medium with glucose as the main carbon source [40,41]. Likewise, present finding regarding the optimal pH for monascus pigments production, the optimal pH for red and orange monascus pigments from *M*. *purpureus* strain MTCC 410 was at 4.5–5.5 [42].

Time is a crucial factor in the industrialization agenda of a given bioprocess. As a rule of thumb, the more reduction is in the overall time to perform a bioprocess, the more reduction is in the capital cost of such bioprocess. The optimal incubation period to achieve the maximal yield of orange and red monascus pigments varied widely from 5 days to 16 days among different strains of *Monascus spp* regarding solid state fermentation or submerged state fermentation [16, 17, 28, 42,43]. These remarkable variations in the incubation time could be assigned to the efficiency of the producer strain and the nature of the components included in the production medium. Easily accessible carbon sources of the fungal strains would guarantee high growth rate and high yields of the pigments as well. At most, simple carbon sources are utilized in submerged state fermentation for monascus pigments production with too short time in due for achieving the highest maximal levels of pigments. In contrast, the usage of too complex, hardly accessible substrates as main carbon sources in solid state fermentation in the course of monascus pigments production would result in too long time in due for achieving the possible maximal levels of pigments.

With regard to aeration in terms of agitation, no available reports in the literature highlighted the effect of agitation on monascus pigments production in solid state fermentation on the bench scale. However, the present enhanced levels of orange and red monascus pigments could be ascribed to the effect of agitation in terms of addition/ distribution of water and improvement in heat transfer allover the flask. Nonetheless, the impact of discontinuous agitation should be tested in prospective studies to reduce the power and the energy required for continuous agitation during the industrialization step.

Pertaining to the amount of corn cob, addition of corn cob grams greater than 24 grams would lead to inhibition of fungal growth and hence a dramatic decrease in the levels of pigments would be noticed. The mechanism of inhibition could be attributed to the extremely low levels of oxygen and uneven distribution of aeration. Conversely, at small amounts of the corn cob less than 24 grams the fungal growth would be restricted to small surface area of the waste. Thus, a notable decrease in the levels of pigments production occurred.

Absence of a perceivable influence of MgSO_4_ and KNO_3_ on the monascus pigments production via by *M*. *purpureus* strain ATCC 16436 may be possibly attributed to the satisfaction of the nutritional requirements of the fungus by both corn cob and glycerol.

The review of literature does address the role of glucose as an efficient, simple, readily accessible carbon source to induce the production of orange and red monascus pigments in both submerged state fermentation and solid state fermentation [41,43]. Conversely, addition of glucose on rice solid state fermentation did not provoke any impact on the levels of monascus pigments production by *M*. *purpureus* MTCC 410 [42]. Although, addition of glycerol and glucose separately on corn cob solid state fermentation did exhibit equivalent, significant, enhanced consequences on monascus pigments production by *M*. *purpureus* strain ATCC 16436. Glycerol, as a low cost, effective monascus pigments inducer, was selected as a co-inducer along with corn cob for monascus pigments instead of glucose. It was reported that high levels of glycerol (>0.5 M) in submerged state fermentation for monascus pigments production did exhibit osmotic stress with low levels of pigments production [11, 23–26]. Unlike previous reports, the present finding does confirm the usage of glycerol at 2.17 M in a co-solid state fermentation with corn cob exhibited enhanced levels of both orange and red pigments by *M*. *purpureus* strain ATCC 16436. Hence, this would reflect the much better tolerance of the fungal strain *M*. *purpureus* strain ATCC 16436 to the osmotic stress enforced by glycerol when compared to previously reported fungal strains affected negatively by glycerol addition to the monascus pigments production.

With regard to inoculum size, low levels of monascus pigments were noticed at low levels of inoculum size of *M*. *purpureus* strain ATCC 16436 and vice versa. Divergence in the optimal inoculum size required to achieve the most likely maximal levels of monascus pigments among various fungal strains could be ascribed to medium composition, growth conditions, environmental stress, and potency of the fungal strains as well.

## Conclusions

In the current study, co-solid state fermentation of the two agro-industrial wastes corn cob and glycerol successfully provoked high levels of orange and red monascus pigments production by *M*. *purpureus* strain ATCC 16436. Meanwhile, the two agro-industrial corn cob and glycerol were efficiently co-valorized. This study does outline a low cost effective, efficient, reproducible, eco-friendly approach towards the co-production of monascus orange and red pigments. Present data would greatly encourage the transfer to the industrialized scale for commercial exploitation of orange and red monascus pigments from *M*. *purpureus* strain ATCC 16436 through the present approach.
